# Glycosylated Hemoglobin as an Age-Specific Predictor and Risk Marker of Colorectal Adenomas in Non-Diabetic Adults

**DOI:** 10.3389/fendo.2021.774519

**Published:** 2021-11-03

**Authors:** Xinyan Yu, Chen Chen, Xiaoxiao Song, Yi Guo, Yuling Tong, Yi Zhao, Zhenya Song

**Affiliations:** ^1^ Department of General Practice and Health Management Center, the Second Affiliated Hospital, School of Medicine, Zhejiang University, Hangzhou, China; ^2^ Department of Big Data in Health Science, School of Public Health, Zhejiang University, Hangzhou, China; ^3^ Center for Biostatistics, Bioinformatics, and Big Data, Second Affiliated Hospital, School of Medicine, Zhejiang University, Hangzhou, China; ^4^ Department of Endocrinology, the Second Affiliated Hospital, School of Medicine, Zhejiang University, Hangzhou, China

**Keywords:** glycosylated hemoglobin (HbA1c), adenomatous polyp, non-diabetic adults, age factor, risk factors

## Abstract

**Background:**

Diabetes is a risk factor for colorectal neoplasms. The association between the level of glycosylated hemoglobin (HbA1c) and the risk of colorectal adenomas (CRAs) in non-diabetic adults needs to be investigated.

**Methods:**

A cross-sectional study was performed on non-diabetic adults with normal HbA1c level who underwent colonoscopy between January 2010 and December 2016 during health check-ups in our hospital in China. The association between HbA1c level and CRAs was assessed by multiple logistic regression models stratified by age group (<40, ≥40 and <50, and ≥50 years old). The age group-specified thresholds for HbA1c on elevated risk of CRAs were estimated using the piecewise logistic regression.

**Results:**

Among the 2,764 subjects, 445 (16.1%) had CRA. The prevalence of CRA varied across the three age groups. A higher HbA1c level was found to be significantly associated with increased CRA risk in the 40–50 years group (odds ratio [OR]=1.70, 95% confidence interval [CI] 1.04–2.78, p=0.035) after adjusting for other related factors, while this association was borderline significant among the 50 years and older group (OR=1.57, 95% CI 0.97–2.54, p=0.067). Based on the piecewise logistic regression analysis results, the thresholds for HbA1c on elevated risk of CRA were 5.44% for the 40–50 years group and 4.81% for the 50 years and older group, respectively.

**Conclusions:**

Higher levels of HbA1c, even within the normal range, were associated with elevated CRA risk among non-diabetic adults. The threshold effects of HbA1c on the risk of CRA varied across different age groups, and early screening colonoscopy might be needed for individuals in their 40s and with HbA1c levels ≥5.44%.

## Introduction

Colorectal cancer (CRC) is one of the most common cancers, with the third highest incidence and the second highest mortality in the world (1). The incidence and mortality of CRC have shown an increasing trend in China over the past decade (2). Understanding the risk factors for CRC can provide guidance for developing strategies targeted toward colorectal screening and its prevention. Several risk factors have been proposed, including age, smoking, alcohol consumption, obesity, high-calorie/fat diets, lack of physical exercise, and family history of CRC (3).

Colorectal adenoma (CRA), which is considered a precursor of most CRCs (4), allows for screening and prevention of CRC by colonoscopy examination and polypectomy, respectively (5). Hyperglycemia plays an important role in the pathogenesis of CRC and CRA (6, 7). A meta-analysis of 17 observational studies showed that type 2 diabetes mellitus is associated with a higher risk of developing CRA (8). Glycosylated hemoglobin (HbA1c) reflects the average blood glucose concentration over the preceding 2–3 months and is a sensitive and reliable marker of abnormal glucose metabolism. Chronic hyperglycemia may result in hyperinsulinemia, and HbA1c may indirectly reflect hyperinsulinemia. Many studies have reported that hyperglycemia and insulin resistance (IR) are associated with an increased risk of CRC (7, 9, 10). However, in contrast to CRC, there are limited data on the relationship between the levels of HbA1c and CRA, and the conclusions are not entirely consistent (6, 11–16). Only a few studies have reported that HbA1c levels are associated with CRAs, but the association between normal HbA1c levels and the risk of CRAs in non-diabetic adults has not been reported (11–14).

The American College of Gastroenterology and Asia Pacific Consensus CRC screening guidelines strongly recommend 50 years as the starting age for screening CRC, while the former conditionally recommends lowering the starting age to 45 years (17, 18). In view of the increasing trend in young-onset CRC worldwide, with newly diagnosed CRC cases increasing to 15% (19, 20), and the rapidly increasing incidence of CRC in patients as early as 40 years of age in China (21), we need to focus on average-risk individuals between 40 and 50 years of age. However, due to the availability of medical resources and national screening programs, CRC screening is less likely to be conducted in average-risk individuals, even in individuals between the ages of 40 and 50 years (22). So far, there is no consensus that persons aged 40–50 years should be screened.

Therefore, the current study aimed to explore the relationship between HbA1c level and the risk of CRAs among different age groups in non-diabetic Chinese adults with normal levels of HbA1c, and focused on identifying high-risk individuals of CRAs in young-onset adults, especially those aged 40–50 years.

## Materials and Methods

### Study Population

**Figure 1:**
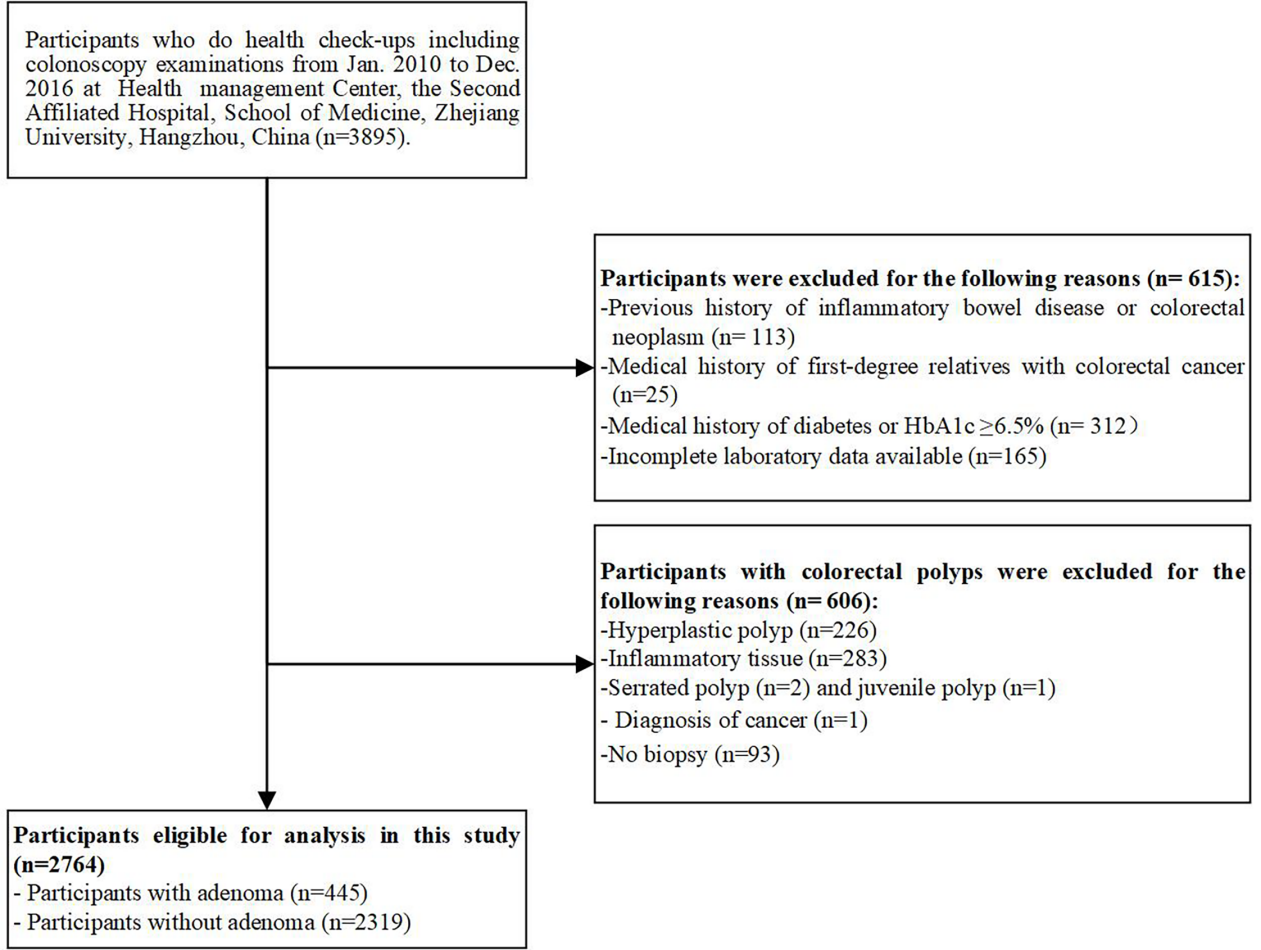
Inclusion and exclusion of study participants.

We conducted a cross-sectional study on a consecutive series of asymptomatic adults who underwent colonoscopy examinations and had a serum HbA1c level determined during routine health check-ups at the Health Management Center, The Second Affiliated Hospital, School of Medicine, Zhejiang University, China, between January 2010 and December 2016. Among the 3895 participants, 615 were excluded for the following reasons: 1) medical history of first-degree relatives with CRC; 2) previous history of inflammatory bowel disease or colorectal neoplasm; 3) medical history of diabetes or HbA1c ≥6.5%; and 4) incomplete laboratory data available. In addition, 606 participants with colorectal polyps were excluded for the following reasons: 1) pathologic diagnosis of a non-adenomatous polyp (i.e., hyperplastic polyps, inflammatory polyps, juvenile polyps, or serrated polyps); 2) pathologic diagnosis of cancer; and 3) without biopsy. After excluding these participants, 445 patients with adenomatous polyps and 2319 polyp-free controls were enrolled in this study (**Figure 1**). The study was performed in accordance with the ethical standards as laid down in the 1964 Declaration of Helsinki and was approved by the Institutional Research Ethics Committee of the hospital (No.20210667), which waived the requirement for informed consent.

### Data Collection

Medical history of hypertension, diabetes, CRC, colorectal polyps, colorectal surgery family history of CRC, and personal history of smoking and alcohol consumption were obtained by trained physicians. Height and weight were measured using an ultrasonic body scale (SK-CK, Shenzhen, China) with subjects standing barefoot while wearing light clothing. Body mass index (BMI) was calculated as weight in kilograms divided by the square of height in meters (kg/m^2^). Blood pressure (BP) was measured using an automatic sphygmomanometer (Omron HBP-9020, Shanghai, China) following a standard protocol after 10 min of rest.

Fasting blood samples were obtained from each participant in the morning after overnight fasting. Total cholesterol (TC), triglyceride (TG), high-density lipoprotein cholesterol (HDL-C), low-density lipoprotein cholesterol (LDL-C), fasting plasma glucose (FPG), and serum uric acid (sUA) levels were analyzed using an automatic chemistry analyzer (Olympus AU4500, Tokyo, Japan) according to the manufacturer’s instructions. White blood cell (WBC) counts were measured using an automatic hematology analyzer (Sysmex XN-9000, Kobe, Japan). Fasting insulin levels were measured using an autoanalyzer (Roche E170, Mannheim, Germany). HbA1c levels were determined using an automatic analyzer (Arkray HA8160, Kyoto, Japan) using standard methods. Insulin resistance (IR) was estimated using the homeostatic model assessment of IR (HOMA-IR): fasting insulin (μU/mL) × FPG (mmol/L)/22.5 (23).

Before colonoscopy examination, participants were prescribed a liquid diet a day before and received 2–3 L of polyethylene glycol solution (HE SHUANG, Shenzhen, China) for bowel preparation. After the bowels were adequately prepared, all colonoscopies were performed by experienced endoscopists with more than ten years of experience. Participants were examined by video colonoscopy (Olympus CF-H290, CF-H260AI, or CF-Q260AI, Tokyo, Japan), after which they underwent total colonoscopy. When colorectal polyps were identified, the endoscopists decided whether biopsy is required as determined by the endoscopists. Each pathological diagnosis was performed by two histopathologists who were unaware of the laboratory test results or medical histories of the subjects.

### Statistical Analysis

Continuous variables are expressed as median and interquartile range, while categorical variables are presented as frequencies with percentages (%). Demographic, CRA status, history of smoking and drinking, anthropometric measurements, and laboratory test results were stratified by age group (<40, ≥40 and <50, and ≥50 years old) and were compared using the Kruskal-Wallis rank test for continuous variables and χ2 test for categorical variables. Univariable logistic regression analysis was performed to examine the unadjusted association between each factor and the presence of CRA for the entire cohort, as well as for each age subgroup. Multiple logistic regression on the effects of related factors on CRA status was applied based on stepwise model selection results to reduce collinearity and overfitting. To account for the rarity of CRA cases (6.52%) in the group aged less than 40 years, the Firth penalized maximum-likelihood method was applied on the logistic regression model for the purpose of bias reduction. The locally weighted scatterplot smoothing (LOWESS) curves were constructed by age groups to visually present the association between HbA1c and the probability of CRA, as well as to explore the potential trend changes of the association. To further investigate the threshold for the effects of HbA1c on the probability of CRA, piecewise logistic regression was performed to identify the breakpoint in each age group. All statistical analyses were performed using Stata/SE 16 (StataCorp, College Station, TX, USA). The Firth penalized maximum-likelihood method and piecewise logistic regression were performed using the user-written Stata programs firthlogit and loghockey, respectively. All statistical significance was set at P<0.05.

## Results

### Characteristics of Subjects

Among the 2,764 subjects in the selected cohort, 445 (16.1%) had CRA. Male subjects accounted for 65.85% of the entire cohort, and the mean age was 45.96 ± 0.19 and 46.56 ± 0.27 years old for males and females, respectively. Demographic and clinical characteristics were compared across the three age groups and are presented in **Table 1**. The prevalence of CRA was significantly different across the three age groups, with the group aged 50 years and older having the highest prevalence (23.97%), followed by the 40–50 years group (14.81%) and the group aged younger than 40 years (6.52%). Characteristics including age, sex, systolic BP (SBP), diastolic DP (DBP), BMI, TC, TG, LDL-C, HDL-C, WBC, FPG, fasting insulin, HbA1c, HOMA-IR, smoking, and medical history of hypertension, varied across the three age groups (P<0.05).

**Table 1:** Characteristics of study subjects by age groups and CRA status.

Variables	<40 years old (n=583)	≥40 and <50 years old (n=1,263)	≥50 years old (n=918)	P value
Subjects with CRA (n, %)	38 (6.52)	187 (14.81)	220 (23.97)	<0.001
Age (years)	36 (33, 38)	44 (42, 47)	54 (52, 59)	0.0001
Male (%)	387 (66.38)	863 (68.33)	570 (62.09)	0.01
Systolic blood pressure (mmHg)	115 (105, 126)	118 (108, 129)	123 (112, 135)	0.0001
Diastolic blood pressure (mmHg)	69 (63, 78)	73 (65, 81)	75 (68, 83)	0.0001
Body mass index (kg/m^2^)	23.06 (20.9, 25.19)	23.98 (22.08, 25.99)	23.92 (21.98, 25.71)	0.0001
Triglyceride (mmol/L)	1.35 (0.86, 2.04)	1.48 (1.05, 2.22)	1.43 (1.05, 2.02)	0.0002
Total cholesterol (mmol/L)	5 (4.49, 5.65)	5.08 (4.47, 5.71)	5.24 (4.64, 5.91)	0.0001
HDL-cholesterol (mmol/L)	1.26 (1.09, 1.51)	1.24 (1.04, 1.48)	1.28 (1.11, 1.48)	0.0031
LDL-cholesterol (mmol/L)	2.84 (2.37, 3.38)	2.9 (2.44, 3.38)	3.04 (2.57, 3.53)	0.0001
White blood cell (x 10^9/L)	6.2 (5.2, 7.3)	6 (5.1, 7.1)	5.7 (4.9, 6.8)	0.0001
Serum urine acid (μmol/L)	351 (272, 413)	350 (280, 415)	341 (283, 395)	0.0845
Fasting insulin (mIU/L)	8.73 (6.22, 12.15)	8.73 (6.14, 12.36)	7.94 (5.59, 11.04)	0.0001
Fasting plasma glucose (mmol/L)	4.83 (4.55, 5.17)	4.94 (4.64, 5.29)	5.10 (4.74, 5.42)	0.0001
Glycosylated hemoglobin (%)	5.4 (5.2, 5.6)	5.5 (5.3, 5.7)	5.7 (5.5, 5.9)	0.0001
HOMA-IR	1.88 (1.32, 2.74)	1.93 (1.33, 2.77)	1.80 (1.20, 2.56)	0.0041
Smoking (%)	141 (24.19)	332 (26.29)	187 (20.37)	0.006
Drinking (%)	112 (19.11)	242 (19.16)	190 (20.70)	0.683
Medical history of hypertension (%)	21 (3.60)	152 (12.03)	189 (20.59)	<0.001

Data are presented as median (P_25_, P_75_) or number (percentage). P values were calculated using Kruskal-Wallis rank test for continuous variables and χ2 test for categorical variables. CRA, colorectal adenoma; HDL, high-density lipoprotein; LDL, low-density lipoprotein; HOMA-IR, homeostatic model assessment of insulin resistance.

### Unadjusted Association Between Potential Influencing Factors and Risk of CRA

Univariable logistic regression analysis was performed to determine the association between clinical factors and CRA status (**Table 2**). For the entire group, all the factors examined were significantly associated with CRA. When stratified by age group, the factors associated with CRA differed across different age groups. For subjects younger than 40 years old, only age, sex, DBP, smoking, and medical history of hypertension were found to be significantly associated with CRA. For the 40–50 years group, age, SBP, DBP, BMI, TC, TG, LDL, WBC, sUA, fasting insulin, HbA1c, and HOMA-IR were all positively associated with increasing CRA risk, while higher HDL levels were associated with lower CRA risk. Within this group, being male, smoking, and drinking were found to be associated with CRA. The results for the 50 years old and over group were similar to those of the 40–50 years group, except that TG, LDL, and drinking were not found to be significantly associated with the risk of CRA.

**Table 2:** Univariable logistic regression analysis on factors associated with colorectal adenomas.

Variables	Overall (n=2,764)		<40 years old ^a^ (n=583)		≥40 and <50 years old (n=1,263)		≥50 years old (n=918)	
	OR (95%CI)	P value	OR (95%CI)	P value	OR (95%CI)	P value	OR (95%CI)	P value
Age	1.07 (1.05-1.08)	<0.001	1.27 (1.10-1.46)	0.001	1.10 (1.04-1.16)	0.001	1.05 (1.02-1.08)	0.001
Male	1.95 (1.53-2.47)	<0.001	2.85 (1.17-6.95)	0.021	1.92 (1.32-2.79)	0.001	2.11 (1.50-2.95)	<0.001
Systolic blood pressure	1.02 (1.01-1.02)	<0.001	1.02 (1.00-1.04)	0.052	1.01 (1.00-1.02)	0.038	1.01 (1.00-1.02)	0.005
Diastolic blood pressure	1.03 (1.02-1.04)	<0.001	1.03 (1.00-1.06)	0.023	1.02 (1.00-1.03)	0.009	1.02 (1.01-1.04)	0.003
Body mass index	1.08 (1.05-1.12)	<0.001	1.05 (0.95-1.15)	0.361	1.08 (1.03-1.14)	0.003	1.08 (1.03-1.14)	0.003
Triglyceride	1.12 (1.06-1.19)	<0.001	1.06 (0.87-1.29)	0.548	1.15 (1.06-1.24)	<0.001	1.14 (1.00-1.30)	0.042
Total cholesterol	1.17 (1.06-1.30)	0.003	0.98 (0.69-1.39)	0.892	1.36 (1.16-1.59)	<0.001	0.97 (0.82-1.14)	0.675
HDL-cholesterol	0.54 (0.39-0.75)	<0.001	0.73 (0.25-2.12)	0.566	0.52 (0.32-0.85)	0.009	0.45 (0.28-0.74)	0.002
LDL-cholesterol	1.21 (1.06-1.38)	0.004	1.10 (0.72-1.67)	0.661	1.39 (1.14-1.69)	0.001	0.99 (0.80-1.21)	0.894
White blood cell	1.13 (1.06-1.21)	<0.001	1.18 (0.99-1.41)	0.067	1.15 (1.04-1.28)	0.006	1.21 (1.10-1.33)	<0.001
Serum urine acid	1.00 (1.00-1.00)	<0.001	1.00 (1.00-1.01)	0.485	1.00 (1.00-1.00)	0.001	1.00 (1.00-1.01)	<0.001
Fasting insulin	1.02 (1.00-1.04)	0.019	1.00 (0.94-1.06)	0.970	1.04 (1.01-1.06)	0.012	1.03 (1.01-1.07)	0.021
Fasting plasma glucose	1.34 (1.12-1.61)	0.001	1.00 (0.52-1.91)	0.993	1.18 (0.88-1.57)	0.274	1.21 (0.93-1.59)	0.163
Glycosylated hemoglobin	2.72 (2.01-3.67)	<0.001	2.23 (0.79-6.27)	0.129	2.28 (1.43-3.65)	0.001	1.67 (1.05-2.64)	0.030
HOMA-IR	1.11 (1.03-1.19)	0.004	1.00 (0.80-1.25)	0.988	1.16 (1.04-1.29)	0.008	1.16 (1.03-1.29)	0.012
Smoking	1.83 (1.47-2.27)	<0.001	2.17 (1.10-4.28)	0.026	2.25 (1.63-3.11)	<0.001	1.63 (1.14-2.33)	0.007
Drinking	1.47 (1.16-1.86)	0.002	1.79 (0.86-3.73)	0.119	1.68 (1.17-2.41)	0.005	1.21 (0.84-1.75)	0.297
Medical history of hypertension	2.28 (1.76-2.94)	<0.001	3.65 (1.17-11.46)	0.026	1.49 (0.97-2.31)	0.069	2.00 (1.41-2.84)	<0.001

a Firthlogit model was applied to reduce bias due to small number of colorectal adenoma cases in subgroup with age younger than 40 years old. HDL, high-density lipoprotein; LDL, low-density lipoprotein; HOMA-IR, homeostatic model assessment of insulin resistance; OR, odd ratio; CI, confidence interval.

### Independent Association Between HbA1c and Risk of CRA

Multiple logistic regression models were constructed for the entire cohort and for each age subgroup based on the stepwise model selection results (**Table 3**). Adjusting for other related factors, increasing HbA1c level was found to be associated with increased CRA risk when all subjects in the cohort were included in the analysis (odds ratio [OR]=1.56, 95% confidence interval [CI] 1.13–2.16, p=0.007). For the youngest group (age<40 years), no association was observed between HbA1c and CRA in both univariable and multiple analyses. For subjects aged 40–50 years, the adjusted analysis results indicated that every 1% increase in HbA1c was associated with a 1.70-fold higher risk of CRA (95% CI 1.04–2.78, p=0.035). In the oldest group, after adjusting for the effects of other influencing factors, HbA1c was borderline significantly associated with CRA (OR=1.57, 95% CI 0.97–2.54, p=0.067).

**Table 3:** Multiple logistic regression analysis on factors associated with colorectal adenomas.

Variables	Overall (n=2,764)		<40 years old ^a^ (n=583)		≥40 and <50 years old (n=1,263)		≥50 years old (n=918)	
	OR (95%CI)	P value	OR (95%CI)	P value	OR (95%CI)	P value	OR (95%CI)	P value
Age	1.07 (1.05-1.08)	<0.001	1.26 (1.09-1.46)	0.002	1.09 (1.03-1.15)	0.003	1.04 (1.01-1.07)	0.008
Male	1.51 (1.15-1.99)	0.003	3.78 (1.26-11.31)	0.017	**-**	**-**	1.85 (1.29-2.64)	0.001
Triglyceride	1.08 (1.01-1.15)	0.023	**-**	**-**	1.10 (1.01-1.19)	0.031	**-**	**-**
Total cholesterol	**-**	**-**	–	–	1.19 (1.00-1.41)	0.051	–	–
White blood cell	1.09 (1.01-1.17)	0.021	1.21 (1.00-1.47)	0.050	**-**	**-**	1.15 (1.04-1.27)	0.008
Serum urine acid	**-**	**-**	0.99 (0.99-1.00)	0.056	**-**	**-**	**-**	**-**
Glycosylated hemoglobin	1.56 (1.13-2.16)	0.007	**-**	**-**	1.70 (1.04-2.78)	0.035	1.57 (0.97-2.54)	0.067
Smoking	1.53 (1.18-1.97)	0.001	**-**	**-**	2.09 (1.50-2.91)	<0.001	**-**	**-**
Drinking	**-**	**-**	**-**	**-**	**-**	**-**	–	–
Medical history of hypertension	1.49 (1.13-1.97)	0.004	2.98 (0.95-9.39)	0.062	**-**	**-**	1.71 (1.19-2.45)	0.004

a Firthlogit model was applied to reduce bias due to small number of colorectal adenoma cases in subgroup with age younger than 40 years old. OR, odd ratio; CI, confidence interval.

### Threshold Effects of HbA1c on Risk of CRA by Age Group

**Figure 2:**
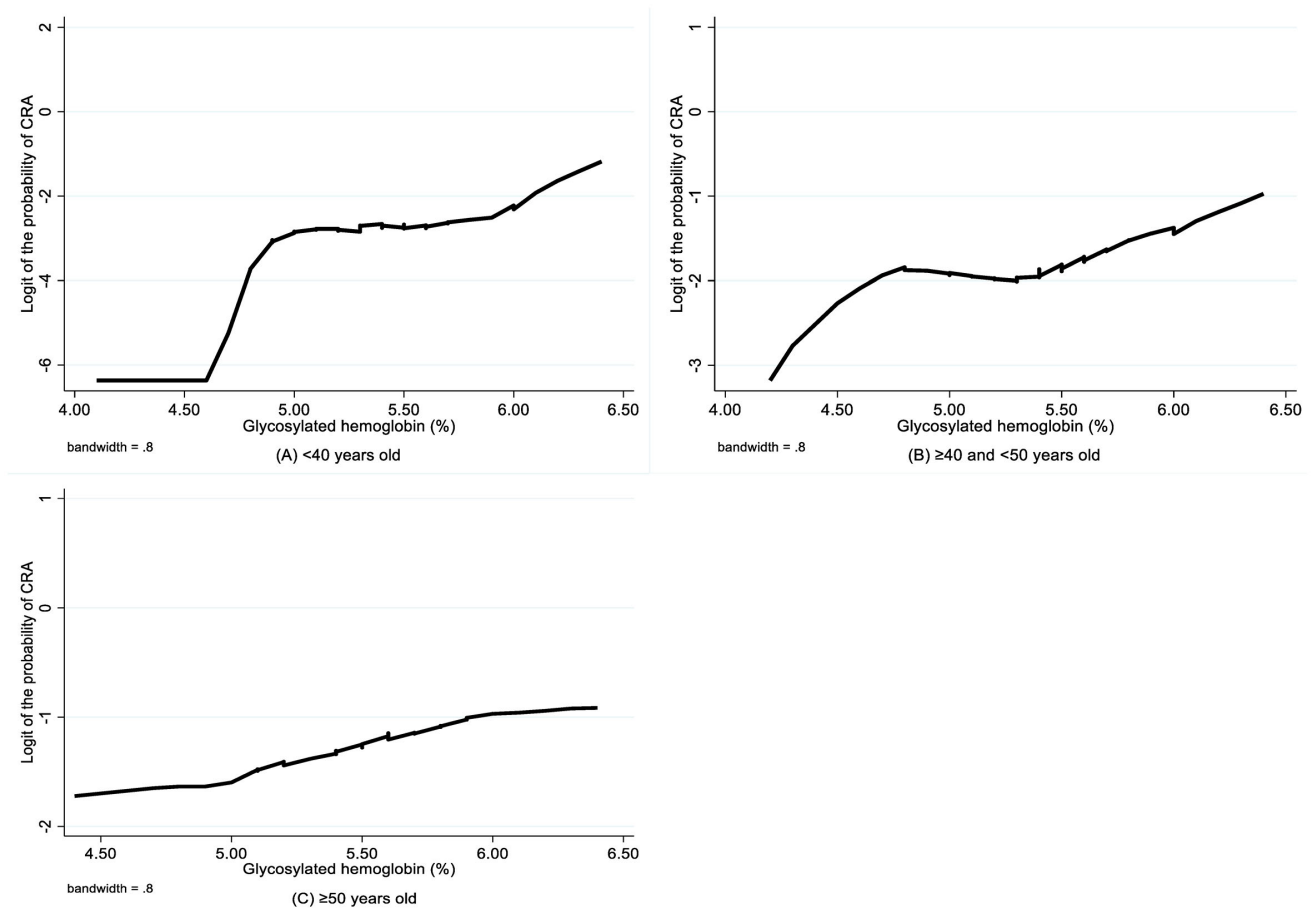
LOWESS smoothing curve on association between glycosylated hemoglobin and CRA by age group. CRA, colorectal adenoma.

The LOWESS smoothing curves exhibited the relationship between HbA1c and logit of the probability of CRA for each age subgroup, which indicated a non-linear relationship, as well as changes in relationship patterns (**Figure 2**). Although HbA1c was found to be not associated with CRA in both unadjusted and adjusted analyses for the youngest group, the CRA risk increased with the increase in HbA1c for the 40–50 years group and the oldest group, and this trend became more evident after a certain breakpoint. Based on the piecewise logistic regression analysis, the breakpoints suggesting thresholds for the effects of HbA1c on the risk of CRA were identified for the 40–50 years group and the oldest group (**Table 4**). When the HbA1c levels were below 5.44% for the 40–50 years group and 4.81% for the oldest group, HbA1c had no significant effect on the risk of CRA; however, above these thresholds, every one unit increase in HbA1c was associated with a 3.17-fold (95% CI 1.40–7.12, p=0.005) and 1.71-fold higher risk (95% CI 1.05–2.78, p=0.030) of CRA in the 40–50 years old group and the oldest group, respectively.

**Table 4:** Piecewise logistic regression results on breakpoint of HbA1c on CRA risk.

	≥40 and <50 years old (breakpoint of HbA1c =5.44%, p<0.001)		≥50 years old (breakpoint of HbA1c =4.81%, p<0.001)	
	< breakpoint	>breakpoint	< breakpoint	>breakpoint
Subjects with CRA (n, %)	62 (11.79)	125 (16.96)	2 (18.18)	218 (24.04)
OR (95% CI, ref: without CRA)	0.96 (0.24 – 3.76)	3.17 (1.40 – 7.12) ^*^	-^a^	1.71 (1.05 – 2.78) ^*^

a Odd ratio was not calculated due to extremely small sample size. ^*^P<0.05.

CRA, colorectal adenoma; HbA1c, glycosylated hemoglobin; OR, odd ratio; CI, confidence interval; ref, reference.

## Discussion

In this study, we found that higher levels of HbA1c were independently associated with an elevated risk of CRAs in 40–50-year-old subjects after adjusting for related factors, whereas among subjects aged 50 years or older, HbA1c levels were only borderline significantly associated with increased risk of CRA. The association between HbA1c and CRAs was not detected in subjects younger than 40 years. Furthermore, we found that HbA1c has a threshold effect on the elevated risk of CRAs, which varied across different age groups. To the best of our knowledge, this study is the first to focus on normal HbA1c levels in non-diabetic adults and stratification by age when exploring the association between HbA1c and CRAs.

Our results support the hypothesis that perturbations in insulin levels and glucose control partially contribute to the etiology of colorectal neoplasms (9, 24). In this study, the results indicated that fasting insulin levels were almost the same in the 40–50 years group and the youngest group, but the levels in the 40–50 years group was considerably higher than those in the oldest group (P<0.001). Similarly, HOMA-IR was the highest in the 40–50 years group, followed by the youngest group and the oldest group successively (P<0.001). Hyperinsulinemia caused by IR is more apparent in 40–50-year-old subjects than in older subjects, since hyperinsulinemia is compromised by pancreatic β-cell dysfunction, which increases with age (25). However, IR was not apparent in the youngest group when compared to subjects aged 40–50 years, which is in agreement with the physiopathology of the earlier stage of hyperglycemia in the youngest group. Meanwhile, in this youngest group, islet function is almost in a compensative normal or high-level state, but the duration of IR has just begun for the younger subjects, whereas the islet function further decreases and may even have insulin deficiency in older subjects. Furthermore, the incidence of CRAs increases with age (26). Therefore, in this study, we speculate that the association between the level of HbA1c and CRAs varied across different age groups.

HbA1c is a reliable marker of hyperglycemia and may be a suitable marker of chronic hyperinsulinemia, as high glucose levels correspond to high insulin levels. Potential mechanisms for the association between hyperglycemia, hyperinsulinemia, and CRA risk have been reported. Hyperglycemia may induce colorectal neoplasia *via* chronic inflammation, oxidative stress, or adipokines (27). Hyperinsulinemia can stimulate the insulin-like growth factor (IGF) system, which plays an important role in cancer development and progression due to increased cell proliferation and anti-apoptotic effects through IGF-I receptor (IGF-IR) over-expression, over-activation, and endocrine/paracrine/autocrine production of its ligands, IGF-I, and IGF-II (28–32). Recently, a prospective cohort study including 397,380 participants provided a strong evidence for the positive association between circulating IGF-I levels and CRC risk (33). A meta-analysis of 19 epidemiological studies revealed that high levels of IGF-I and IGF-II significantly increased the risk of CRC (34).

Our results showed a significant association between HbA1c level and the risk of CRAs among non-diabetic adults with normal HbA1c levels, and this association varied among the different age groups. We also detected a threshold effect of HbA1c on the risk of CRA. When the HbA1c level exceeded 5.44% for subjects in their 40s or 4.81% for subjects aged 50 years and older, the risk of CRA significantly increased. The HbA1c threshold was not present among the group younger than 40 years old; however, since we only examined the effect of HbA1c within the normal range, we cannot exclude the possibility that there might also be a threshold effect in the younger group, but this is outside the scope of the study. Although our result was consistent with multiple recent studies, these studies did not focus on normal HbA1c levels in non-diabetic adults and were not stratified by age group. Two cross-sectional studies with subjects aged >40 years found that HbA1c >6.0% for non-diabetic adults and that HbA1c ≥6.5% was independently associated with CRA (13, 14). A cross-sectional study of 819 asymptomatic men aged 40–59 years found that higher levels of HbA1c were associated with a higher risk of CRA in those aged 40–50 years (11). A large-scale longitudinal study with 5,289 asymptomatic subjects aged >30 years revealed that HbA1c levels were significantly associated with the occurrence of CRA detected during surveillance colonoscopy (12). In contrast, some studies are inconsistent with the findings of this study. Two retrospective studies with diabetic subjects found no significant association between HbA1c level and CRA or high-risk CRA (15, 16). Meanwhile, a cross-sectional study with 19,361 asymptomatic subjects found that increasing levels of HbA1c were significantly associated with the prevalence of CRA, but there was no evidence of an association between HbA1c and CRA after adjusting for related risk factors (6). Discrepancies among these studies may result from different study designs, the composition of the study population, and the degree of control for potential confounders.

We also showed that there was a significant relationship between FPG and CRA in the whole population, but this relationship was not significant within each age group. Further analysis showed that there was no significant relationship between FPG and CRA (OR=1.13, 95% CI 0.94–1.37, P=0.194) after controlling for age, which implies that the influence of FPG on CRA was mainly attributed to the age effect. However, there was still a significant association between HbA1c and CRA (OR=1.79, 95% CI 1.30–2.50, P<0.001) in the whole population even after controlling for age, indicating that HbA1c still influenced the risk of CRA regardless of the influence of age. In the multiple analysis, after adjusting for other related factors, this association remained significant for subjects 40–50 years of age and was borderline significant for subjects in the oldest group. Therefore, we inferred that HbA1c may be a more effective predictor or risk marker for CRA than FPG. Consistent with our findings, Hsu et al. reported that HbA1c, compared to FPG, is more strongly and independently associated with colorectal neoplasia, even in non-diabetic participants (35). Our findings add to the evidence that HbA1c is superior to FPG in assessing the risk of complications associated with chronic hyperglycemia.

This study, however, is subject to limitations. First, we did not have data on postprandial blood glucose and postprandial insulin, so we cannot completely evaluate the blood glucose spectrum; otherwise, we can better evaluate the association between the level of HbA1c and CRA. Second, due to the nature of the cross-sectional design, the causal relationship between HbA1c level and the outcome of CRAs could not be established. A prospective study is needed to further examine the role of HbA1c in the pathological mechanisms of CRAs. Finally, the generalization of our results should be interpreted with caution. Since the subjects in the study were visitors of a health management center, they might be more concerned about their health than the general population.

In conclusion, higher HbA1c levels, even within the normal range, were associated with elevated CRA risk among non-diabetic adults, and this association varied among the different age groups. Furthermore, we found that there is an age group-specific threshold for HbA1c on elevated risk of CRA, and subjects aged 40–50 years with HbA1c level ≥5.44% were high-risk individuals for CRA. Therefore, we suggest that combining HbA1c level and age stratification might be necessary for non-diabetic adults when developing a plan for CRC, and early screening colonoscopy might be needed for subjects in their 40s when their HbA1c level is ≥5.44%. Currently, the ages between 40 to 50 years have no score in the revised risk-stratified scoring system of the Asia Pacific Consensus on CRC (17), and future insights will lead to an improved risk-stratified scoring system by adopting a more individualized approach to CRC screening.

## Data Availability Statement

The raw data supporting the conclusions of this article will be made available by the authors, without undue reservation.

## Ethics Statement

The studies involving human participants were reviewed and approved by the Institutional Research Ethics Committee of the Second Affiliated Hospital, School of Medicine, Zhejiang University (No. 20210667). The ethics committee waived the requirement of written informed consent for participation.

## Author Contributions

XY: conception, design, interpretation, and drafting of the manuscript. CC: data analysis, interpretation, and drafting of the manuscript. XS: critical review. YG: critical review. YT: critical review. YZ: critical review. ZS: conception, design, and critical review. All authors contributed to the article and approved the submitted version.

## Conflict of Interest

The authors declare that the research was conducted in the absence of any commercial or financial relationships that could be construed as a potential conflict of interest.

## Publisher’s Note

All claims expressed in this article are solely those of the authors and do not necessarily represent those of their affiliated organizations, or those of the publisher, the editors and the reviewers. Any product that may be evaluated in this article, or claim that may be made by its manufacturer, is not guaranteed or endorsed by the publisher.
